# Doctors and Nurses: A Systematic Review of the Risk and Protective Factors in Workplace Violence and Burnout

**DOI:** 10.3390/ijerph18063280

**Published:** 2021-03-22

**Authors:** Jose Miguel Giménez Lozano, Juan Pedro Martínez Ramón, Francisco Manuel Morales Rodríguez

**Affiliations:** 1 Department of Educational and Developmental Psychology, Faculty of Psychology, Campus Universitario de Cartuja, University of Granada, 18071 Granada, Spain; josemi1992@correo.ugr.es; 2 Department of Developmental and Educational Psychology, Campus of Espinardo, University of Murcia, 30100 Murcia, Spain; juanpedromartinezramon@um.es

**Keywords:** burnout, workplace violence, protective factors, risk factors

## Abstract

The present study aims analyze the risk factors that lead to high levels of burnout among nurses and physicians and the protective factors that prevent them. Thus, it is also intended to explore the possible correlation between physical and verbal violence produced at work and the symptoms derived from burnout. Methods: The search was carried out on the Scopus, PubMed and Web of Science databases between 2000 and 2019 (on which date the bibliographic search ends). Descriptive studies estimating the prevalence of workplace violence and risk and protective factors and burnout were included. An adapted version of the Downs and Black quality checklist was used for article selection. 89.6 percent of the studies analysed were in the health sector. There is a significant correlation between burnout symptoms and physical violence at work. On the one hand, the risk factors that moderate this correlation were of structural/organisational type (social support, quality of the working environment, authoritarian leadership, little autonomy or long working days, etc.) and personal type (age, gender, nationality or academic degree, etc.). On the other hand, protective factors were the quality of the working environment, mutual support networks or coping strategies. The results were analysed in-depth and intervention strategies were proposed.

## 1. Introduction

Workplace violence (WV) is understood as any type of act, incident, or behavior in which a person is abused, threatened, humiliated, or assaulted in the workplace, including verbal and physical assaults [1]. The National Institute of Safety and Hygiene in the Workplace (Spanish acronym: INSHT) affirms that WV is one of the main health risk factors for those who are active in the workplace, since such actions result in a multitude of psychological and emotional conditions that prevent the development of a ‘working life or aggravate the problems that occur regardless of work. The many studies related to WV have observed that the risks of exposure to this type of situation are very high in those jobs that imply providing a service to people, so the nursing and medical profession would stand out for the nature of one’s own work as a facilitator for the creation of WV situations [2]. The WV is characterized by being diverse in its ways of expressing itself, physically (hitting, pushing, kicking…) verbally (yelling, insults, offensive comments in general…) or sexually [3].

Previous models related to working conditions and the work environment assumed that workplace violence was based exclusively on situations involving physical violence, leaving aside stress, overwork, or the psychological demands of the task itself [4]. Since then, many authors have focused their work on discovering the causes and consequences that it occurs ‘daily’. For example, Mucci [5] observed that working conditions such as high work demands, job control, type of leadership, peer support, company organization, and even gender (higher among women than among men) correlated with WV. Following the same line of research, but focusing on individual factors, Robelski [6] showed that emotional demands, job satisfaction, and each person’s own resilience had a direct influence on WV. On the other hand, the consequences generated by violence at work are related to the high levels of stress experienced. Thus, the vast majority of studies have found that workers who have repeatedly suffered some type of aggression at work present high levels of anxiety, depression, generalized fear, insomnia or emotional problems that lead to more serious disorders such as Post-Traumatic Stress Disorder or burnout [7].

The concept of burnout has been closely related to the adverse effects produced by the labor sector since 1970 [8], and is usually defined as “a frequent physical and emotional exhaustion of workers, especially those who provide some type of service to others, resulting from the conditions and risk situations experienced.” Maslach and Jackson were the first to define burnout. They developed an instrument to assess the burnout that occurred in three areas using the Maslach Burnout Inventory (MBI) [9] and which is composed of three dimensions: (1) Emotional Exhaustion (EE) related to mental and emotional fatigue, (2) Depersonalization, related to the most negative behaviors of individuals, (3) and low Personal Accomplishment (PA), meaning a tendency to be evaluated negatively based on job performance [10]. Subsequently, in 1992, the International Statistical Classification of Diseases and Related Health Problems define the burnout under the heading “Problems related to life management difficulty” (Z73) and is defined as a “State of vital exhaustion” (ICD-10, 2012) [11]. The American Psychiatric Association (APA) does not yet include a definition of burnout in its Diagnostic and Statistical Manual—V (DSM-V, 2013) [12]. This disorder is often characteristic of the most stressful jobs, which involve constant direct contact with other people such as police officers, teachers and especially health sector employees such as nurses, doctors, assistants, etc. [12]. Burnout has consequences on the physical and mental health of nurses, workplace; for example, this may affect the individual, generating physical symptoms of fatigue, anxiety, sleep disorders, insomnia, headaches, and frequent colds alongside reduced concentration and memory [13], also affecting work levels, such as absenteeism or intention to leave [14].

Nurses have been considered a risk group because of the high levels of burnout they suffer daily [15]. For example, it has been observed that nurses would have a much higher prevalence of burnout than other sectors [16] due to frequent understaffing in hospitals that increases the nurse-to-patient ratio, high work overload, long working days and long working hours that vary from day to day [17]. In addition, it has been observed that health sector personnel, especially those working in higher intensity facilities such as those involving emergencies, palliative care, or ambulance services, are noted for a high percentage of aggressions either by a patient or a colleague [18]. These assaults can be verbal or physical, although sexual assaults on female health workers are also very common [19].

On the other hand, doctors are another group to consider due to the high prevalence shown by the results of several investigations. A systematic review by Rotenstein, Torre, Ramos [20] of 182 articles showed a prevalence of 0% to 86.2% in mental exhaustion among physicians, 0% to 89.9% in depersonalization, and 0% to 87.1% in low personal accomplishment.

The aim of this work is to analyze the risk factors that lead to high levels of burnout levels among nurses and physicians, and the protective factors that can help prevent burnout.

## 2. Materials and Methods

A bibliographic review has been carried out following PRISMA’s recommendations for descriptive and systematic reviews.

### 2.1. Search Strategies

During the second and third quarters of 2020, an exhaustive search was carried out in the databases of Scopus, Pubmed, WOS, PsycINFO and Cochrane. A free search was carried out in Google Scholar and in portals related to occupational health and healthcare at work to identify those studies that were not published in the databases (13.2%). The search strategy was based on the combination of specific search terms: Violence [Mesh], Workplace Violence [Mesh], Health Personnel [Mesh], Burnout, protective factors, risk factors, medical staff.

### 2.2. Data Extraction

Data extraction was performed using a standard data extraction form developed by the *Joanna Briggs Institute Reviewers’ Manual for The Systematic Review of Prevalence and Incidence Data* [21]. We used a specific coding manual to extract information from the primary studies. To ensure rigor in the final selection of studies, the three reviewers ran the form through all of the articles to check their suitability, relevance and precision.

### 2.3. Inclusion Criteria

**Figure 1:**
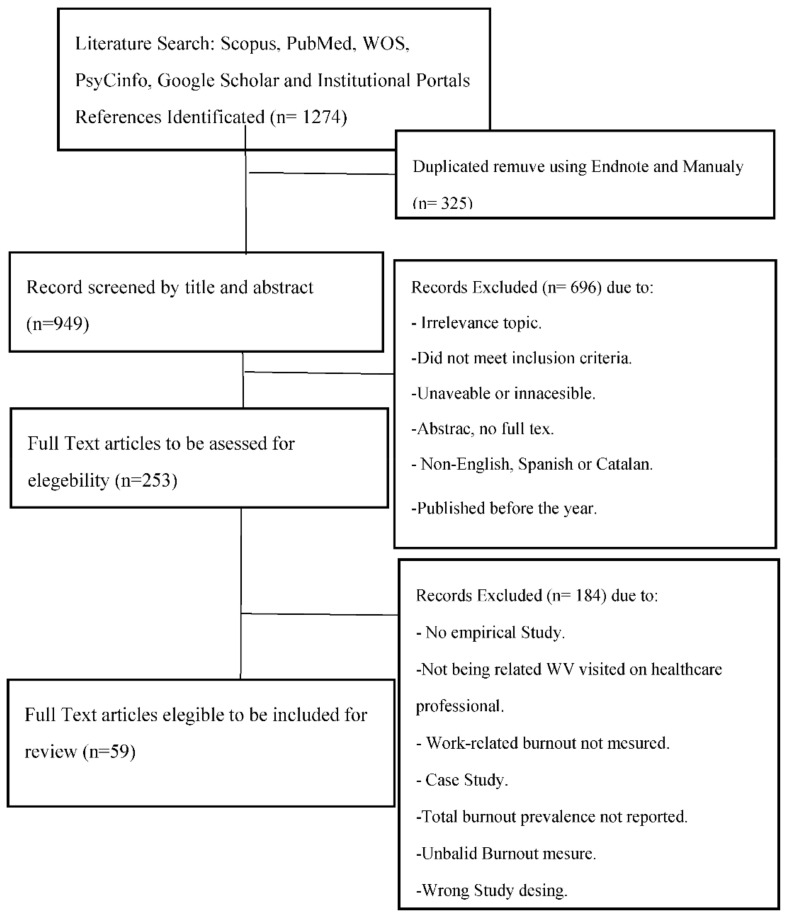
PRISMA flow Chart.

Studies were included according to the following criteria: (a) empirical; (b) published between 2000 and 2019 (on which date the bibliographic search ends); (c) empirical studies; (d) published in Spanish, Catalan or English; (e) the articles must evaluate t burnout syndrome with the standardized Maslach Burnout Inventory questionnaire [22]; (f) compare burnout levels in medical and nursing professionals; (g) evaluating at least one type of WV (physical, verbal or sexual); (h) aiming to observe the protective and risk factors related to WV. Articles of an informative nature, experts’ opinions published in editorials and letters to the editor were excluded (Figure 1). The diagram was made following the PRISMA declaration [23].

### 2.4. Selection of the Studies

The main search yielded a result of 949 posts. The first selection phase was carried out based on reading the titles and abstracts of the publications; 253 articles were selected for further study. The exclusion criteria in this first phase were: non-empirical studies, not published before the year 2000, not written in Spanish, Catalan or English, not being directly related to workplace violence towards health professionals and not evaluating burnout with the MBI. The selection was then examined by means of a complete reading of the articles taking into account the inclusion criteria. Finally, 59 documents were selected for their final review. 13.2% of the final selection came from Google Scholar and institutional portals.

### 2.5. Data Analysis Section

Variables and information extracted from the studies. Literature search results and data extraction results were summarized descriptively. To exclude duplicate articles, we used the program Endnote for manual selection. A summary of efficacy outcomes was presented based on the different outcome measures, controls and interventions. A narrative synthesis was therefore generated, considering the total number of studies that reported results, the methodological quality, and the quality of evidence for the outcomes to yield final conclusions. The general characteristics of systematic reviews extracted were used as exploratory variable PRISMA scores. We included: number of authors, nurses versus physicians, percentage of women versus men, transversal studies versus other methodological interventions, type of burnout and WV instrumentals used in the study, presence of risk and protectives factors versus absence. If the value of these factors was *p*-value < 0.05, it was considered statistically significant for the systematic review.

## 3. Results

The results of the final selection of the studies are show on Table 1 (See Table 1). The information in Table 1 allowed us to calculate the statistics. The descriptive results are shown on Table 2 (See Table 2). The total population analysed was 22,993 (*N* = 22,993), with a mean age of 34.18 (*Mean*= 34.18; *SD* = 6.14). A higher women’s employment rate can be observed in the world health sector, representing 61.43% (*N* = 14,126) of all studies, men being at 38.56% (*N* = 8867). Most of the research related to burnout and WV is focused on the nursing profession, with 71.64% (*N* = 16,473) representing the nurses who have been evaluated. Doctors appear to take up 28.49% (*N* = 6551) of the research, and a lower percentage is taken up by nursing assistants, orderlies and/or technicians, 1.16% (*N* = 269). The most evaluated specialties are the ICU or Intensive Care Unit (Critical Care) (*N* = 9344; % = 40.63%), Emergencies (*N* = 6719; % = 29.22%), the Mental Health specialty (*N* = 4236; % = 18.54%) and Surgery (*N* = 4187; % = 18.45%). In terms of work contracts, those of a fixed or full-time nature are the most frequent, 57.66% (*N* = 13,258), as are, to a lesser extent, part-time—28.07% (*N* = 6456)—and temporary contracts—14.02 (*N* = 3279). The university academic level is most common among the population examined, 38.02% (*N* = 8793), as is experience of between 5 and 15 years, 42.11% (*N* = 9683).

Table 3 (See Table 3) shows the results of the mean of the different levels that make up burnout.

Regarding gender, we can observe that women in the health sector appear to suffer from higher burnout levels than men. For example, women ($\bar{x}$ = 28.2; *SD* = 6.0) seem to have higher levels of EE compared to men ($\bar{x}$ = 24.3; *SD* = 6.5) and the same hold with DP, in which women get a score of ($\bar{x}$ = 12.2; *SD* = 4.2), while that for men is ($\bar{x}$ = 11.7; *SD* = 4.2). The PA subcategory is the only variable in which men ($\bar{x}$ = 24.3; *SD* = 7.2) obtain a higher score than women ($\bar{x}$ = 25.4; *SD* = 7.2). Regarding the professional category, it is nurses who obtain the worst results in terms of EE ($\bar{x}$ = 28.1; *SD* = 6.1) to DP ($\bar{x}$ = 11.9; *SD* = 4.0) and PA ($\bar{x}$ = 25.1; *SD* = 6.7). In the specialty area, there are three categories with very similar values, including the Intensive Care Unit (ICU) with an EE of ($\bar{x}$ = 29.1; *SD* = 6.4), a DP of ($\bar{x}$ = 12.8; *SD* = 3.9) and a PA of ($\bar{x}$ = 27.7; *SD* = 6.3). The Emergency specialty has an EE of ($\bar{x}$ = 29.6; *SD* = 6.1), a DP of ($\bar{x}$ = 10.8; *SD* = 4.1) and a PA of ($\bar{x}$ = 25.4; *SD* = 7.3). Similar values were found in the Mental Health specialty, with an EE of ($\bar{x}$ = 28.0; *SD* = 6.5), a DP of ($\bar{x}$ = 12.1; *SD* = 3.9) and a PA of ($\bar{x}$ = 28.6; *SD* = 5.9).

Table 4 (See Table 4) shows the main characteristics of violence and the protective and risk factors that promote it.

The vast majority of the studies analysed (*N* = 65; % = 90.2%) show that the most frequent causal agent endured by healthcare professionals is the patient; to a lesser extent, family members or partners (*N* = 26; % = 36.1%); and finally, coworkers (*N* = 11; % = 15.2%). The most common type of violence, as collected by all the investigations, are verbal (*N* = 72; % = 100%), including shouting, insults or threats. Workers claim that at some time in their working life they have suffered some type of physical violence (*N* = 57; % = 79.1%) and, to a lesser extent, a sexual assault (*N* = 23; % = 31.9%). Regarding risk and protective factors, it should be clarified that any action contrary to a risk factor, or that goes in the opposite direction, is understood as a protective factor. Thus, the factors were classified into two series of categories, the structural/organizational type, and the personal type. For example, within the former group, 97% (*N* = 70) of the studies analysed found that social support from coworkers is one of the risk and protective factors that most affect workplace violence in the healthcare sector; this would also be highly influenced by family support (*N* = 35; % = 48.6%). A determining factor seems to be the professionals’ type of contract, full-time (*N* = 48; % = 66.6%) or part-time (*N* = 36; % = 52%). The quality of the work environment (*N* = 17; % = 23.6%), the type of leadership of the plant manager (*N* = 22; % = 30.5%) and the level of autonomy (*N* = 9; % = 12.5) are among the other risk and protective factors found. On the other hand, personal factors are those related, for example, to workers’ job satisfaction (*N* = 70; % = 97.2%), coping strategies (*N* = 28; % = 38.8%), self-efficacy (*N* = 12; % = 16.6%) or empathy (*N* = 11; % = 15.2%).

## 4. Discussion

The objective of this systematic review is to observe the levels of WV and burnout in the healthcare sector and to verify the most influential risk and protective factors.

The analysis of the results of the different studies finally selected shows that there is a high percentage of professionals who at some time in their working lives have been exposed to some type of WV, whether verbal, physical, or sexual. This, in turn, appears to affect the high levels of observed burnout, which are higher among women than among men, although this may also be due to the large female representation in the total sample. Following in this same line, according to the Labour Force Survey (LFS) developed by the National Institute of Statistics (INE, from its Spanish initials) in Spain, with data from the last quarter of 2018, women appear to hold 71.7% of jobs in the healthcare sector [82]. Among many other variables, the burnout of professionals is mainly influenced by their specialization, type of work employment and their experience within the field. When it comes to the type of contract, full-time employment involves more effort on the part of the worker and requires greater attitudinal resources to carry out the work required, these cause greater emotional exhaustion (EE), lower feelings of personal accomplishment (PA) and therefore more negative thoughts about their competency (DP) [28].

In relation to the profession among nurses and doctors that suffers the greatest WV, the nursing profession faces the most exposure. This is due, in large part, to the fact that they are directly in contact with patients. They are, therefore, the first exposed to complicated situations that require a lot of control and that generate great emotional wear. Various studies have shown that it would be the nurses who, due to the situations of experienced violence, would obtain the worst levels of burnout and emotional intelligence. This would translate into high levels of anxiety, lack of self-esteem, insomnia problems, depression or even physical effects such as a higher percentage of those suffering from cancer. [83]

The specialties in which higher levels of burnout are contemplated are actually those in which patients are in the most serious conditions and which require more need to manage unpredictability in the workplace, as can happen in the Intensive Care Unit (ICU), in the emergency room, or in the mental health area, which involves unpredictable patients with low levels of self-control [84]. These studies corroborate that high levels of burnout in specializations where the patient’s life may be in danger correlate with a high percentage of WV [85], mainly from the patients, family members or partners. It can be seen that despite the pressure affecting nurses, doctors and nursing assistants, among others, there is not a high percentage of attacks on the part of coworkers.

There are many risk factors that make WV more likely. If one examines them in-depth, what is striking is that those with the most impact are related to high levels of effort (structural and/or organizational factors) and workers’ lack of self-control and self-regulation skills (personal factors). Two types of factors were categorized for a better understanding, but neither should be understood separately, but rather as a set that affects and influences everyone equally. For example, a healthcare professional who has a full-time work contract is more likely to be exposed to WV, since this entails greater emotional exhaustion, and more exposure to extreme situations and to all types of people with multiple reactions. We understand, in accordance with the results, that this probability of experiencing WV will increase if he or she works within a specialty in which moments of great tension and stress are experienced. However, in relation to this equation, various types of factors, both organizational and personal, can considerably reduce the WV suffered, such as a healthy work environment, high levels of social support from colleagues, or even possessing intrinsic coping strategy, self-efficacy and adaptation resources. These, in turn, would increase job satisfaction and therefore reduce the frequency of WV [86], causing burnout levels to decrease drastically, with the health benefits this environment entails [87].

It is here, therefore, that the role of institutions becomes important. Creating intervention programmes that aim to alleviate levels of WV should be a priority. For example, the Zero Tolerance Policy is a political campaign, spread all over the world, a benchmark in the United Kingdom that includes several organizations such as the British Columbia Occupational Health and the Safety Agency for Healthcare and the National Health Service (NHS) [30], and addresses the issue of violence against healthcare workers on the part of co-workers and beneficiaries. This campaign provides healthcare personnel with the necessary resources, such as courses or workshops and the creation of support networks within the community, and promotes awareness in relation to patients to show the reality of this matter. For this, a positive work culture must be created where all those involved (workers and patients) treat each other with respect, with a focus on positive work recognition and conflict resolution [88]. The application of these types of policies can have a great benefit for workers, such as a more positive responses to stressful situations [61].

Another very similar program was the Assaulted Staff Action Program (ASAP), which aimed to identify “high-risk” patients (with a criminal record or previous reports of high levels of aggression) and to give workers suitable warning prior to the visit. According to some studies, it was shown that WV decreased significantly when using this method [88]. In that sense, it is important to note that workplace violence is one of the possible causes of burnout in the health sector [81].

Therefore, this is not merely an internal struggle on the part of workers when facing patients. Government agencies should also be the main stakeholders and work alongside other parties to reduce such incidents, as the quality of the healthcare sector depends on the satisfaction of nurses, doctors and other specialized employees.

### Limitations and Future Research

The present systematic review has some limitations. The inclusion and exclusion criteria were clearly defined, but more research into a more specific database such as CINAHL (Cumulative Index to Nursing & Allied Health Literature) would be advised in the case whereby an article only appears in it and not in WOS. There is also the risk of an article included in the so-called gray literature (doctoral theses, etc.) that was not located.

It would be interesting in future studies to focus on examining other variables considered relevant in the selected studies on psychoeducational intervention programs in which this instrument is applied. In this sense, this study can provide useful information to researchers and professionals for decision-making in this thematic line. More longitudinal research is required focused on evaluating the effectiveness of programs for the prevention of burnout and that not only focus on studying the effects of burnout on healthcare workers but also, for example, in the university environment. In addition, despite the importance of improving well-being and quality of life, there are several policies that could be sought to reduce the WV they experience throughout their professional lives.

## 5. Conclusions

It can be concluded that workplace violence is one of the possible causes of burnout in the health sector.

The risk and protective factors were classified into two series of categories, the structural/organizational type (for example, social support from coworkers, family support, professionals’ type of contract, the quality of the work environment) and the personal type (for example, workers’ job satisfaction, coping strategies, self-efficacy, empathy, etc.). Regarding risk and protective factors, it should be clarified that any action contrary to a risk factor, or that goes in the opposite direction, is understood as a protective factor.

As we can see, there are many studies that have studied the effects of burnout on workers in the health sector, but there are few action policies that aim to reduce the WV they experience throughout their professional lives. We must emphasize that this would imply not only action from within the healthcare sector but more re-training and building awareness in society about the importance of such work, and to provide enough information to control all kinds of uncontrolled impulse in emergency situations.

We can point out the importance of institutions promoting recommendations and policies that can develop empathy and a positive work culture where those involved treat each other with respect, recognition, and work to prevent workplace violence, work stress and improve the quality of life and well-being of its members.

## Figures and Tables

**Table 1:** Description of the included studies.

Autores	*n*	Age	% Women	Type Work	Type Study	Burnout Evaluation	Workplace Violence Evaluation	Otras Medidas
(Out, 2005) [24]	385	41	97.2%	Nurses	Transversal	-Maslach Burnout Inventory-Human Services Survey (MBI-HSS)	-The Negative Acts Questionnaire (NAQ). -Specific Events in the Workplace (Keashly, Trott, & MacLean, 1994).	Job Satisfaction Subscale of the Ward Organisational Features Scales (WOFS). -Turnover Cognitions Scale (Bozeman & Perrewe, 2001). -World Assumptions Scale (WAS; Janoff-Bulman, 1989): Evalúa los factores relacionados con la benevolencia. -Symptom Assessment—45 Questionnaire (SA-4): Evalúa aspectos psicológicos.
(Merezc, 2006) [25]	413	38	X	Physicians	Transversal	-Maslach Burnout Inventory (MBI)	Own questionnaire on assaults suffered at work.	-General Health Questionnaire (GHQ-28)
(Isaksson, 2008) [26]	196	41.5	85.7%	Assistants	Transversal	-Maslach Burnout Inventory-Human Services Survey (MBI-HSS)	Own questionnaire on assaults suffered at work.	-Memories of Parental Rearing -Temperament and Character Inventory (TCI-125)
(Merecz, 2009) [27]	1554	39.54	X	Nurses	Transversal	-Maslach Burnout Inventory (MBI)	-Workplace Aggression Questionnaire (EWAQ). -Direct reactions to aggressive behaviours (DRAB).	-Job satisfaction: 22-itemdeveloped by the Work Psychology Department of NIOM. -Well-being psychological: General Health Questionnaire (GHQ-28).
(Spence-Laschinger, 2010) [28]	415	27.24	94%	Nurses	Transversal	-Maslach Burnout Inventory (MBI)	-Negative Acts Questionnaire-Revised (NAQ-R)	-The Authentic Leadership Questionnaire (ALQ) (Avolio, Gardner, & Walumbwa, 2007). -Psychological Capital Questionnaire (Luthans et al., 2007)
(Dikmetas, 2011) [29]	270	30	34.07%	Physicians	Transversal	-Maslach Burnout Inventory (MBI)	-Leymann Inventory of Psychological Terror: Evalúa el bullying y el mobbing	Demographic and professional background.
(Happell, 2011) [30]	123	41.38	X	Nurses	Transversal	-Maslach Burnout Inventory (MBI)	-Minnesota Satisfaction Questionnaire (MSQ).	-Survey of Nurses’ Attitudes to Seclusion Survey (SNASS; Heyman 1987). -Elsom Therapeutic Optimism Scale (ETOS; Elsom & McCauley-Elsom 2008)
(Murillo, 2011) [31]	20	31	45%	Physicians	Transversal	-Maslach Burnout Inventory (MBI-HSS)	-Protocol of assaults on doctors that evaluates data on the assault	Demographic and professional background.
(Spence Laschinger, 2011) [32]	165	28.28	93.2%	Nurses	Transversal	-Maslach Burnout Inventory (MBI)	Negative Acts Ques- tionnaire-Revised (NAQ-R)	-The Areas of Worklife Scale (AWS). -Psychological Capital Questionnaire (PCQ). -Pressure Management Indicator(PMI)
(Patrick, 2011) [33]	339	28.10	91.5%	Nurses	Transversal	-Maslach Burnout Inventory (MBI)	Negative Acts Ques- tionnaire-Revised (NAQ-R)	-The Authentic Leadership Ques-tionnaire (ALQ) -Job Satisfaction scale.
(Sundin, 2011) [34]	1216	42.37	94.3%	Nurses	Longitudinal	-Maslach Burnout Inventory (MBI)	-Work Environment Survey (SWES)	-Work and social support: Items extracted from the Swedish Work Environment Survey (SWES).
(Hensel, 2012) [35]	926	39.7	82.3%	Nurses, physicians and assistants	Transversal	-Maslach Burnout Inventory-Human Services Survey (MBI-HSS)	-Questionnaire on the frequency of assaults suffered at work.	-Demographic and professional background.
(Gascon, 2012) [36]	1826	41.84	64.2%	Nurses, physicians and assistants	Transversal	Maslach Burnout Inventory (MBI-HSS)	Workplace violence Questionnaire (Gascón et al. 2009b)	-Areas of Work-life Scale (AWS, Leiter & Maslach 2004a)
(Pranjic, 2012) [37]	116	X	72.4%	Nurses, physicians and assistants	Transversal	-Maslach Burnout Inventory (MBI)	-Mobbing Questionnaire	-Job satisfaction questionnaire. -Work commitment
(Roldán, 2012) [38]	315	43.92	37.4%	Physicians	Transversal	-Maslach Burnout Inventory (MBI)	-Questionnaire on the frequency of assaults suffered at work.	-The Beck Depression Inventory (BDI). -The Beck Anxiety Inventory (BAI).
(Spence-Laschinger, 2012) [39]	205	X	92%	Nurses	Transversal	-Maslach Burnout Inventory (MBI)	-Questionnaire on the frequency of assaults suffered at work.	-Work satisfaction (Shaver & Lacey, 2003).
(Galián-Muñoz, 2013) [40]	137	42	82.5%	Nurses and assistants	Transversal	-Maslach Burnout Inventory (MBI)	Hospital Aggressive Behaviour Scale-Users	-General Health Questionnaire GHQ-28
(Read, 2013) [41]	342	28.1	91.5%	Nurses	Transversal	-Maslach Burnout Inventory (MBI)	-Negative Acts Questionnaire— Revised (NAQ-R) -Workplace Incivility Scale (Cortina et al. 2001).	Conditions of Work Effectiveness Questionnaire II (Laschinger et al. 2001) Career Turnover Intentions (Kelloway et al., 1999) -Areas of Worklife Scale, Community subscale (Leiter and Maslach, 2004) -Areas of Worklife Scale, Values subscale (Leiter and Maslach, 2004) -Areas of Worklife Scale, Fairness subscale (Leiter and Maslach, 2004) -Psychological Capital Questionnaire (Leiter and Maslach, 2004) -Authentic Leadership Questionnaire (Luthans et al., 2007) -Pressure Management IndicatorPhysical Symptoms subscaleEnergy Levels subscale (Williams and Cooper, 1998)
(Pineau-Stamr, 2013) [42]	205	28.1	91.5%	Nurses	Longitudinal	-Maslach Burnout Inventory (MBI)	-Negative Acts Questionnaire— Revised (NAQ-R)	-Job Turnover Intentions Scale, (Kelloway, Gottlieb, y Barham’s 1999). -Authentic Leadership Questionnaire (ALQ)
(Threadgill, 2013) [43]	185	X	86%	Nurses	Transversal	-Maslach Burnout Inventory (MBI)	-Negative Acts Questionnaire—Revised (NAQ-R)	-Demographic and professional background. -intention to quit
(Hensel, 2014) [44]	671	38.7	83.0%	Assistants	Transversal	Maslach Burnout Inventory-Human Services Survey (MBI-HSS)	-Staff Observation Assessment Scale—revised (SOAS-R). -Questionnaire on the frequency of assaults suffered at work.	-Emotional reactions to aggressive behaviour scale. -Difficult behaviour self-efficacy scale. -Positive Work Motivations Scale (PWMS) and General Positive Contributions Scale (GPCS).
(Hu, 2014) [45]	424	X	96%	Nurses	Transversal	-Maslach Burnout Inventory (MBI)	Own questionnaire on assaults suffered at work.	-Demographic and professional background.
(Trépanier, 2014) [46]	699	43.99	90.5%	Nurses	Longitudinal	-Maslach Burnout Inventory General Survey (MBI-GS)	Negative Acts Questionnaire— Revised (NAQ-R)	-Satisfaction: Work-Related Basic Need Satisfaction scale
(Waschgler, 2014) [19]	694	42	83.4%	Nurses	Transversal	-Maslach Burnout Inventory (MBI)	Overall Job Satisfaction	-General Health Questionnaire GHQ-28.
(Abdo, 2015) [47]	550	31.0	72.3%	Nurses and Physicians	Transversal	-Maslach Burnout Inventory (MBI)	Own questionnaire on assaults suffered at work.	-Job satisfaction questionnaire
(Alameddine, 2015) [48]	915	X	79.4%	Nurses	Transversal	-Maslach Burnout Inventory (MBI)	-Exposure and consequences of violence: verbal abuse and physical violence in the last 12 months	-Demographic and professional background. -Intention to quit
(Dal-Pai, 2015) [49]	269	48.5	73%	Nurses and Physicians	Transversal	-Maslach Burnout Inventory (MBI)	-Survey Questionnaire Workplace Violence in the Health Sector	-Self-Report Questionnaire (SRQ-20)
(Menon, 2015) [50]	99	40	50%	Nurses and Physicians	Transversal	-Maslach Burnout Inventory (MBI)	-Descriptive questionnaire on working conditions and violence suffered.	Work stress evaluation questionnaire.
(Pintado-Cucarella, 2015) [51]	29	39.24	65.5%	Physicians	Transversal	Maslach Burnout Inventory-Human Services Survey (MBI-HSS)	Own questionnaire on assaults suffered at work	Jefferson Medical Empathy Scale
(Viotti, 2015) [52]	630	37.97	82.2%	Nurses and Assistants	Transversal	-Maslach Burnout Inventory (MBI)	-Customer-Related Social stressors (CSS) inventory	-Labor resources.
(Deniz, 2016) [53]	120	29.47	46.7%	Nurses and Physicians	Transversal	-Maslach Burnout Inventory (MBI)	Own questionnaire on assaults suffered at work.	Demographic and professional background.
(Galián-Muñoz, 2016) [54]	1489	42.09	82.7%	Nurses, physicians and assistants	Transversal	-Maslach Burnout Inventory (MBI)	The Hospital Aggressive Behaviour Scale—Users (HABS-U).	-Job satisfaction.
(Llor-Esteban, 2016) [55]	518	41.3	86.3%	Nurses	Transversal	-Maslach Burnout Inventory—General Survey (MBI-GS).	Own questionnaire on assaults suffered at work	-Overall Job Satisfaction Scale (OJS).
(Anwar, 2017) [56]	286	28.9	X	Nurses	Transversal	-Maslach Burnout Inventory (MBI)	Own questionnaire on assaults suffered at work.	Demographic and professional background.
(Bawakid, 2017) [57]	246	35	57.3%	Physicians	Transversal	-Maslach Burnout Inventory (MBI)	Own questionnaire on assaults suffered at work.	Demographic and professional background.
(Karsavuran, 2017) [58]	454	55	32.0%	Physicians	Transversal	-Maslach Burnout Inventory (MBI)	Own questionnaire on assaults suffered at work.	-Demographic and professional background. -Leymann Inventory of Psychological Terrorization (LIPT).
(Hamdan, 2017) [59]	444	30	76.8%	Nurses and Physicians	Transversal	-Maslach Burnout Inventory (MBI)	Own questionnaire on assaults suffered at work.	Demographic and professional background.
(Portoghese, 2017) [60]	40	X	75.0%	Physicians	Transversal	-Maslach Burnout Inventory-Human Services Survey (MBI-HSS)	-Fear of future violent events at work (Rogers and Kelloway, 1997). -Psychological aggression (Rogers and Kelloway, 1997).	-Demographic and professional background. -Job Control
(Rafeea, 2017) [61]	100	35	66%	Nurses, Physicians and Assistants	Transversal	-Maslach Burnout Inventory (MBI)	Own questionnaire on assaults suffered at work	-Demographic and professional background.
(Wongtongkam, 2017) [62]	48	39.19	64.8%	Nurses	Transversal	-Abbreviated Maslach Burnout Inventory.	Workplace violence: personal experience of the last 30 days, causes and contributing factors. Authors’ characteristics	Available resources. Impact of event scale-revised (IES-R). Evaluate Post-Traumatic Stress.
(Copeland, 2018) [63]	147	35	63%	Nurses and physicians	Transversal	-Maslach Burnout Inventory (MBI)	Own questionnaire on assaults suffered at work.	-Job Satisfaction
(Coskun-Cenk, 2018) [64]	143	X	58.7%	Nurses and physicians	Transversal-descriptive	-Maslach Burnout Inventory (MBI)	Own questionnaire on assaults suffered at work.	Demographic and professional background.
(Kim, 2018) [65]	170	X	75%	Nurses	Transversal	-Maslach Burnout Inventory (MBI)	Own questionnaire on assaults suffered at work.	AACN’s HWE Questionnaire: Evaluates collaboration between colleagues, communication skills, effectiveness of decision-making, leadership, recognition
(Looff, 2018) [66]	114	35.2	59%	Nurses	Transversal	-Maslach Burnout Inventory (MBI)	-Modified Overt Aggression Scale (Oliver, Crawford, Rao, Reece, & Tyrer, 2007).	-Dutch Bar-On Emotional Quotient Inventory. Bar-On 2006): Evaluate Emotional Intelligence. -NEO Five-Factor Inventory (NEO-FFI): Evaluate personality factors. -Demands and Support questionnaire: Assess emotional stress.
(Shier, 2018) [67]	674	42.91	86.6%	Nurses, Physicians and Assistants	Transversal	-Maslach Burnout Inventory (MBI)	Own questionnaire on assaults suffered at work.	-Relationships -Intention to Leave—A further adaptation to Kline and Graham (2009): intention to quit work. -Life Scale (SWLS): Satisfaction with life.
(Zaczyk, 2018) [68]	74	40	90.5%	Nurses	Transversal	-Maslach Burnout Inventory (MBI)	Own questionnaire on assaults suffered at work	Demographic and professional background.
(Aguilar-Nájera, 2019) [69]	411	X	X	Physicians	Transversal	-Maslach Burnout Inventory (MBI)	Own questionnaire on assaults suffered at work	Demographic and professional background.
(Ajoudani, 2019) [70]	278	33.76	85.8%	Nurses	Transversal	-Maslach Burnout Inventory (MBI)	-Negative Acts Questionnaire (NAQ)	-Moral Distress Scale-Revised
(Akram, 2019) [71]	350	42.6	40%	Physicians	Transversal	Maslach Burnout Inventory—General Survey (MBI-GS).	-Counterproductive Work Behavior -Abusive supervisor.	-Labor resources.
(Andela,2018) [72]	481	39	96%	Nurses	Transversal	-Maslach Burnout Inventory (MBI)	Elderly abuse scale developed by Huguenotte (2012).	-Job stressors and job resources were measured scale.
(Ghaziri, 2019) [73]	95	44	75%	Nurses	Transversal	-Maslach Burnout Inventory (MBI)	-Own questionnaire on assaults suffered at work.	Demographic and professional background.
(Castro Negreiros, 2019) [74]	96	X	X	Assitants	Transversal	-Maslach Burnout Inventory (MBI)	-Test Escala Cisneros.	Demographic and professional background.
(Goussinsky, 2019) [75]	105	37.7	84%	Nurses	Transversal	-Maslach Burnout Inventory (MBI)	-Own questionnaire on assaults suffered at work.	-Coworker support Sub-scale: Peer support. -Occupational Coping Self-EfficacyQuestionnaire for Nurses (OCSE-N): Self-efficacy strategies.
(Jiménez, 2019) [76]	565	36	37.4	Nurses and Physicians	Transversal	-Maslach Burnout Inventory (MBI)	-Own questionnaire on assaults suffered at work.	Demographic and professional background.
(Looff, 2019) [77]	110	35.5	59%	Nurses	Longitudinal	-Maslach Burnout Inventory (MBI)	Questionnaire on the frequency of violence suffered at work.	Dutch Bar-On Emotional Quotient Inventory. Bar-On 2006): Evaluate Emotional Intelligence. -NEO Five-Factor Inventory (NEO-FFI): Evaluate personality factors. -Demands and Support questionnaire: Assess emotional stress.
(Kim, 2019) [78]	324	X	96.3%	Nurses	Transversal	-Maslach Burnout Inventory (MBI). -Professional Quality of Life (ProQOL) Scale (Stamm 2009).	-Negative Acts Questionnaire-Revised (NAQ-R)	Turnover intention
(Rayan, 2019) [79]	118	29.14	56%	Nurses	Transversal	-Maslach Burnout Inventory (MBI)	-Own questionnaire on assaults suffered at work.	The Perceived Stress Scale
(Yasar-Hacer, 2019) [80]	310	35.4	52.3%	Physicians	Transversal	-Maslach Burnout Inventory (MBI)	-Own questionnaire on assaults suffered at work.	Demographic and professional background.
(Vincent-Höper, 2020) [81]	582	X	80%	Nurses	Transversal	-Maslach Burnout Inventory (MBI)	-Own questionnaire on assaults suffered at work.	Demographic and professional background.

**Table 2:** Descriptive results.

Variable	Categories	*N* (=22,993)	%
Gender			
	Women	14,126	61.43
	Men	8867	38.56
Profession			
	Nurses	16,473	71.64
	Physicians	6551	28.49
	Assistants	269	1.16
Specialty			
	ICU	9344	40.63
	Emergency	6719	29.22
	Mental Health	4236	18.54
	Surgery	4187	18.45
	Paediatrics	2080	9.04
	Internal Medicine	1552	6.74
	Admissions	915	3.97
Type of employment			
	Full-Time	13258	57.66
	Part-Time	6456	28.07
	Temporary	3279	14.2
Academic level			
	University degree	8793	38.02
	Vocational training/technicians	7512	32.67
	Secondary school certificate	6642	28.88
	PhD	46	0.20
Experience			
	Less than 5 years	6751	29.36
	5–15 years	9683	42.11
	15–30 years	4598	19.99
	More than 30 years	1961	8.52

**Table 3:** Results of the mean of the different levels that make up burnout.

Variable	Category	*Mean* (*SD*) of the Subscales Created by the MBI		
		EE	DP	PA
Gender				
	Men	28.2 (6.0)	12.2 (4.2)	24.3 (7.2)
	Women	24.3 (6.5)	11.7 (4.2)	25.4 (7.2)
Profession				
	Nurses	28.1 (6.1)	11.9 (4.0)	25.1 (6.7)
	Physicians	26.3 (5.4)	12.0(4.3)	24.7 (6.7)
Specialty				
	ICU	29.1 (6.4)	12.8 (3.9)	27.7 (6.3)
	Emergency	29.6 (6.1)	10.8 (4.1)	25.4 (7.3)
	Mental Health	28.0 (6.5)	12.1 (3.9)	28.6 (5.9)
	Surgery	27.7 (6.7)	12.6 (4.1)	27.4 (5.8)
	Paediatrics	24.1 (6.4)	11.1 (3.8)	24.6 (5.2)
	Internal Medicine	26.3 (6.6)	10.2 (3.3)	26.8 (6.7)
	Admissions	24.4 (6.8)	9.8 (4.1)	1.9 (3.9)
Type of employment				
	Full-time	28.3 (7.4)	12.8 (4.1)	28.4 (6.3)
	Part-time	24.1 (6.8)	10.2 (4.4)	23.2 (4.6)
	Temporary	27.6 (7.1)	11.9 (3.9)	25.6 (6.9)
Experience				
	Less than 5 years	28.8 (6.3)	12.2 (4.0)	23.5 (6.3)
	5–15 years	28.2 (6.3)	11.3 (3.3)	25.4 (7.7)
	15–30 years	25.9 (6.5)	12.7 (4.3)	25.2 (6.4)
	More than 30 years	26.4 (6.7)	11.7 (4.2	23.5 (6.4)

MBI, Maslach Burnout Inventory; EE, Emotional Exhaustion; DP, Depersonalization; PA, Personal Accomplishment.

**Table 4:** Main characteristics of the violence suffered at workplace and the protective and risk factors.

Variable	Categories	Subcategories	*N =* 72	%
Causal Agent				
	Patient		65	90.2%
	Partner/Family Members		26	36.1%
	Coworkers		11	15.2%
Type of violence				
	Verbal		72	100%
	Physical		57	79.1%
	Sexual		23	31.9%
Risk and protective factors				
	Organizational/structural			
		Full-Time	48	66.6%
		Part-Time	36	52%
		Social Support	70	97.2%
		Family Support	35	48.6%
		Quality of the work environment	17	23.6%
		Leadership	22	30.5%
		Level of Autonomy	9	12.5%
		Resource Access	25	34.7%
	Personal			
		Job satisfaction	70	97.2%
		Empathy	11	15.2%
		Adaptation	7	9.7%
		Coping strategies	28	38.8%
		Self-efficacy	12	16.6%

## Data Availability

The data could be requested by the scientific community in the ethical terms to be determined.
